# The TAL Effector PthA4 Interacts with Nuclear Factors Involved in RNA-Dependent Processes Including a HMG Protein That Selectively Binds Poly(U) RNA

**DOI:** 10.1371/journal.pone.0032305

**Published:** 2012-02-22

**Authors:** Tiago Antonio de Souza, Adriana Santos Soprano, Nayara Patricia Vieira de Lira, Alexandre José Christino Quaresma, Bianca Alves Pauletti, Adriana Franco Paes Leme, Celso Eduardo Benedetti

**Affiliations:** Laboratório Nacional de Biociências (LNBio), Centro Nacional de Pesquisa em Energia e Materiais (CNPEM), Campinas, SP, Brazil; University of Kansas Medical Center, United States of America

## Abstract

Plant pathogenic bacteria utilize an array of effector proteins to cause disease. Among them, transcriptional activator-like (TAL) effectors are unusual in the sense that they modulate transcription in the host. Although target genes and DNA specificity of TAL effectors have been elucidated, how TAL proteins control host transcription is poorly understood. Previously, we showed that the *Xanthomonas citri* TAL effectors, PthAs 2 and 3, preferentially targeted a citrus protein complex associated with transcription control and DNA repair. To extend our knowledge on the mode of action of PthAs, we have identified new protein targets of the PthA4 variant, required to elicit canker on citrus. Here we show that all the PthA4-interacting proteins are DNA and/or RNA-binding factors implicated in chromatin remodeling and repair, gene regulation and mRNA stabilization/modification. The majority of these proteins, including a structural maintenance of chromosomes protein (CsSMC), a translin-associated factor X (CsTRAX), a VirE2-interacting protein (CsVIP2), a high mobility group (CsHMG) and two poly(A)-binding proteins (CsPABP1 and 2), interacted with each other, suggesting that they assemble into a multiprotein complex. CsHMG was shown to bind DNA and to interact with the invariable leucine-rich repeat region of PthAs. Surprisingly, both CsHMG and PthA4 interacted with PABP1 and 2 and showed selective binding to poly(U) RNA, a property that is novel among HMGs and TAL effectors. Given that homologs of CsHMG, CsPABP1, CsPABP2, CsSMC and CsTRAX in other organisms assemble into protein complexes to regulate mRNA stability and translation, we suggest a novel role of TAL effectors in mRNA processing and translational control.

## Introduction

Plant pathogenic bacteria have developed sophisticated mechanisms to suppress defenses and modulate transcription of host plants to cause disease. Such mechanisms usually involve the transfer of the so-called bacterial type-III effectors to the interior of the plant cell by the type-III secretion system [1]. The transcriptional activator-like (TAL) effectors of the AvrBs3/PthA protein family are good examples of bacterial proteins that are targeted to the nucleus of plant cells to manipulate gene expression [2]. These proteins have the ability to activate transcription in host and non host plants through the recognition of specific promoter regions of target genes [3]–[6]. The interaction of a TAL effector with its target DNA is mediated by the repeat domain of the protein, which comprises a variable region made of nearly identical tandem repeats of 34 amino acids that define the DNA specificity [7]–[9].

In the interaction of citrus plants with *Xanthomonas citri*, the causal agent of citrus canker, it has been shown that the TAL effector protein PthA is not only required for canker elicitation but sufficient to promote cell hypertrophy [10]–[14]. However, while target genes and DNA specificity of TAL effectors have been elucidated in great detail in the past few years [2], [15], how TAL effectors control transcription in the host is not yet clear.

To address this question, and considering that the activity of TAL effectors as transcriptional activators would likely depend on the action of host nuclear factors, we performed numerous two-hybrid screenings of a sweet orange (*Citrus sinensis*) cDNA library using different variants of PthAs as baits. Initial screenings using PthAs 2 and 3 as baits revealed a number of interactions with citrus proteins implicated in nuclear import, transcriptional regulation and DNA repair mechanisms [16]. Among the isolated proteins, a citrus protein complex comprising a cyclophilin (CsCYP), a thioredoxin (CsTDX) and the CsUEV/UBC13 heterodimer, involved in K63-linked ubiquitination and DNA repair, was characterized [16]. Surprisingly, while all PthA variants strongly interacted with the sweet orange importin-α, required for their nuclear import, PthAs 1 and 4 interacted weakly with the citrus protein complex compared to PthAs 2 and 3 [16]. This fact suggested that the PthA variants, although highly homologous to each other, have preferential protein targets in citrus cells.

In line with this idea, and to gain further insights into the mode of action of PthA proteins as transcription factors, we performed two-hybrid screening using PthA4 as bait, which is considered the main PthA variant required for canker elicitation [12], [14]. Here, we describe new interacting partners of PthA4 and show that all of them are homologous to nuclear factors involved in chromatin remodeling and repair, transcriptional regulation and mRNA stabilization/modification. Additionally, we show that the majority of the PthA4 interactors recognize other PthA variants and interact with each other, indicating the existence of an as yet uncharacterized citrus multiprotein complex. In this work, we characterize in more detail the features of one of the components of this multiprotein complex, a high-mobility group protein (CsHMG) that associates with all PthA variants and is homologous to the Arabidopsis HMGB1 involved in cell growth [17].

Although HMG proteins are known to play important roles as DNA-bending transcriptional factors by facilitating the recruitment and assembly of nuclear proteins involved in chromatin remodeling, transcriptional regulation and DNA repair [18]–[22], they were recently shown to bind branched RNA molecules and to possibly participate in mRNA processing [23]. We show here that, in addition to binding to double strand DNA, CsHMG selectively binds to poly(U) RNA, a property that is novel among HMG-box proteins. Furthermore, we surprisingly found that PthA4 also selectively binds to poly(U) RNA and that both CsHMG and PthA4 interact with two poly(A)-binding proteins (PABP1 and 2), which are connected to the citrus multiprotein complex via interactions with a structural maintenance of chromosomes protein (CsSMC) and a translin-associated factor X (CsTRAX). Thus, the results shown here suggest that CsHMG and PthA4 may play roles beyond that of an architectural DNA-bending factor and transcriptional activator, respectively, including mRNA stabilization and processing.

## Materials and Methods

### Yeast two-hybrid assays

Yeast two-hybrid screenings were performed using the PthA4 protein cloned into the pOBD vector as bait and a *C. sinensis* leaf cDNA library cloned into the pOAD vector as prey [16]. The initial screening was performed on synthetic complete medium lacking tryptophan, leucine and histidine (SC -Trp -Leu -His). Isolated colonies were picked and subsequently grown for 5 days at 30°C on SC lacking Trp, Leu, His and adenine (SC -Trp -Leu -His -Ade). pOAD plasmids recovered from positive clones were sequenced and, when required, the full-length citrus cDNAs were obtained by reverse-transcription PCR and subcloned downstream of and in frame with the fusion proteins Gal4AD (pOAD), Gal4BD (pOBD), GST (pGEX-4T1) and 6xHis (pET28). The invariable leucine-rich repeat (LRR) domain of PthAs was amplified by PCR and subcloned into the *Nde*I/*Not*I sites of pOBD and into the *Sal*I/*Not*I sites of pGEX4T-1.

Protein-protein interactions were further verified by yeast two-hybrid assays using baits (pOBDs) and full-length preys (pOADs), including controls (empty pOBD+pOAD-prey and pOBD-bait+empty pOAD), as previously described [16]. The cells were grown on SC -Trp -Leu -His in the presence or absence of adenine and containing 0, 3 or 5 mM 3-aminotriazole (3AT) for 5 days at 30°C.

### Protein purification and GST pulldown

The full-length 6xHis-tagged PthAs (1 to 4) and a derivative carrying 5.5 repeat units plus the C- terminus (PthA5.5rep+CT) were expressed in *Escherichia coli* BL21(DE3) cells and purified by affinity chromatography, as described previously [16]. Prey proteins and the PthA LRR were subcloned into pGEX4T1 and expressed in BL21(DE3) cells upon IPTG induction for 3 h at 30°C. Cell pellets were suspended in PBS buffer containing 1 mM DTT and lysozyme (1.0 mg/ml). After sonication and centrifugation, soluble fractions of GST fusions were immobilized on glutathione resin and non-bound proteins were removed with four PBS washes. Approximately 50 µg of the 6xHis-tagged proteins were incubated with the resins containing GST and GST-fusions for 2 h at 4°C. The beads were washed four times with PBS then eluted with reduced glutathione buffer. Eluted fractions were resolved on 10% and 13% SDS-PAGE gels. Proteins were transferred onto nylon membranes, probed with the anti-PthA (1∶5000), anti-CsHMG (1∶3000) or anti-GST (1∶3000) sera and developed with the ECL kit (GE Healthcare).

### CsHMG purification and antibody production

The full-length CsHMG, its HMG-box domain only (CsHMGΔNΔC) or the N- (CsHMGΔN) and C- (CsHMGΔC) terminal truncated derivatives were expressed in BL21(DE3) cells grown at 37°C in LB supplemented with kanamycin (50 µg/mL) to an OD_600 nm_ = 0.6, followed by induction with 0.4 mM IPTG for 3 h. Cells were harvested by centrifugation, suspended in binding buffer (140 mM NaCl, 2.7 mM KCl, 10 mM Na2HPO4, 1.8 mM KH2PO4, pH 7.4) and incubated on ice with lysozyme (1.0 mg/ml) and sonicated. Clarified supernatants were loaded on a HiTrap chelating HP column (GE Healthcare). Eluted fractions were concentrated, treated with DNaseI (10 µg/mL) and RNase A (10 µg/mL) for 20 min at 4°C, and loaded on an ionic-exchange HiTrap heparin column. Protein fractions were eluted with phosphate buffer (20 mM phosphate, 50 mM NaCl, pH 6.6) and analyzed by SDS-PAGE. Purified CsHMG (∼1 mg) was used to immunize rabbits for anti-serum production.

### CsHMG detection in plant cell extracts

Six-month-old plants of sweet orange (*C. sinensis*) were obtained from certified nurseries and kept in a growth room at 25–28°C under 14 h/day fluorescent light. Etiolated epicotyls of sweet orange ‘Hamlin’ were obtained according to de Oliveira et al. [24]. Seeds of *Arabidopsis thaliana* Col-1 and T-DNA insertion line SAIL261_B02, corresponding to the heterozygous mutant *hmgb-1* [17], were purchased from ABRC and grown in soil in a growth room at 18–22°C under a 16 h/day light regime. Plant materials were frozen and ground in liquid nitrogen and the powder was suspended in lysis buffer (20 mM Tris-HCl, 100 mM NaCl pH 7.2) under slow agitation at 4°C. Cell debris and insoluble materials were separated by centrifugation at 5000 rpm at 4°C and the soluble fractions were analyzed by 13% SDS-PAGE gels and transferred onto nylon-membranes for Western-blot detection.

Sweet orange leaves were ground in phosphate-buffered saline, pH 7.4, containing 10 mM MgCl, 0,05% Triton X-100, 10 mM EDTA and 0.1 mM PMSF. The suspension was cleared by centrifugation and soluble proteins were incubated overnight at 4°C, under agitation, with the purified PthA2 and 4-GST fusions, or GST alone, immobilized on glutathione resins. Resins were washed four times with 20 resin volumes of extraction buffer at 4°C and bound proteins were resolved on 13% polyacrylamide SDS gels and probed with the anti-CsHMG serum.

### Mass spectrometry analysis

Young leaves of sweet orange were macerated in lysis buffer (20 mM Tris-HCl pH7.4, 15 mM imidazole, 25 mM NaCl, 10% glycerol, 0,05% Triton X-100, 0.1 mM PMSF) and cleared by centrifugation. Purified 6xHis-tagged CsSMC and CsTRAX, immobilized on a cobalt resin, were incubated with the citrus cell lisates overnight at 4°C, under agitation. The resins were washed four times with 20 resin volumes of lysis buffer at 4°C and bound proteins were resolved on 10% polyacrylamide SDS gels. Silver-stained bands were cut, reduced, alkylated and digested with trypsin. The resulting peptide mixtures were reconstituted in 0.1% formic acid and analyzed on an ETD enabled Orbitrap Velos mass spectrometer (Thermo Fisher Scientific) connected to nanoflow liquid chromatography (LC-MS/MS) by an EASY-nLC system (Proxeon Biosystem) through a Proxeon nanoelectrospray ion source. Peptides were separated on a 2–90% acetonitrile gradient in 0.1% formic acid using a pre-column EASY-Column (2 cm×id 100 µm, 5 µm particle size) and an analytical column EASY-Column (10 cm×id 75 µm, 3 µm particle size) at a flow rate of 300 nl/min over 20 min. The nanoelectrospray voltage was set to 1.7 kV and the source temperature was 275°C. All instrument methods for the Orbitrap Velos were set up in the data dependent acquisition mode. The full scan MS spectra (m/z 300–2000) were acquired in the Orbitrap analyzer after accumulation to a target value of 1e^6^. Resolution was set to *r* = 60,000 and the 20 most intense peptide ions with charge states ≥2 were sequentially isolated to a target value of 5,000 and fragmented in the linear ion trap by low-energy CID (normalized collision energy of 35%). The signal threshold for triggering an MS/MS event was set to 1000 counts. Dynamic exclusion was enabled with an exclusion size list of 500, exclusion duration of 60 s, and repeat count of 1. An activation q = 0.25 and activation time of 10 ms were used. Peak lists (mgf) were generated from the raw data files by the software Mascot Distiller v.2.3.2.0, 2009 (Matrix Science Ldt.) and searched against the citrus EST (>200.000 sequences) and citrus genome (13.000 unigenes) databases using engine Mascot v.2.3.01 (Matrix Science Ltd.), with carbamidomethylation as fixed modifications, oxidation of methionine as variable modification, one trypsin missed cleavage and a tolerance of 10 ppm for precursor ions and 1 Da for fragment ions. Only peptides with a minimum of five amino acid residues which showed significant threshold (p<0.05) in Mascot-based score were considered in the analysis.

### Electrophoretic mobility shift assays (EMSA)

EMSA was performed using 40 pmoles of double-stranded DNA from the citrus *pr5* promoter or derived from the multiple-cloning site of the pBluescript plasmid vector (Stratagene). Purified full-length CsHMG (100 to 500 ng) was incubated on ice with the DNA fragments in binding buffer (20 mM Tris-HCl, 100 mM NaCl, 10% glycerol e 1 mM EDTA, pH 7.5) for 15 min. Complexes were resolved on TBE-buffered non-denaturing 6% polyacrylamide gels and visualized by ethidium bromide staining. For the RNA-protein interactions, the oligoribonucleotides A-20, C-20, G-20, U-20 or the 5′-UUAUUAUUUAUUUAUUUAUU-3′ probe were labeled with ^32^P using 1 U of T4 PNK (Fermentas) and 20 µCi of [γ^32^P]-ATP. Labeled probes were purified and incubated for 20 min with the full-length or truncated forms of CsHMG or PthA4 (100 to 500 ng) in 20 µL reactions in binding buffer containing 20 mM Tris-HCl, 100 mM NaCl, 10% glycerol e 1 mM EDTA, pH7.5 (for CsHMG) or in 12 mM Tris-HCl, 60 mM KCl, 1 mM DTT, 2.5% glycerol, 5 mM MgCl_2_, 0.2 mM EDTA and 0.05% NP-40, pH 7.5 (for PthA4). RNA-protein complexes were resolved in non-denaturing 13% polyacrylamide gels, for CsHMG-RNA complexes, or 6% polyacrylamide gels, for PthA4-RNA complexes, and exposed to radiographic films for visualization. For CsHMG, the binding mixes were also cross-linked in a UV-crosslinker (Stratagene) for 5 min to form stable RNA-protein complexes. The samples were loaded in a denaturing 10% polyacrylamide gel and detected by autoradiography.

## Results

### Identification of DNA and RNA-binding proteins as PthA4 interactors

To extend our knowledge on the mode of action of PthA proteins as eukaryotic transcriptional modulators, yeast two-hybrid screenings of a sweet orange cDNA library [16] were performed using the PthA4 variant as bait. The majority of the isolated prey proteins could be classified into only three major functional categories including chromatin remodeling and repair, transcriptional regulation and RNA stabilization/modification. Table 1 lists the PthA4-interacting proteins implicated in chromatin structure, DNA repair and mRNA regulatory processes for which yeast two-hybrid and GST pulldown assays were confirmed (see below).

**Table 1:** *Citrus sinensis* proteins identified as targets of PthA4 are homologous to nuclear factors involved in chromatin remodeling and repair, transcription regulation and mRNA stabilization/modification.

Protein	Accession	Features	Predicted biological function	PthA interactor	References
CsHMG	JN600529	DNA binding; HMG-box domain	chromatin remodeling; DNA repair; transcription control	1, 2, 3, 4	[17]–[22], [34]–[39]
CsPABP1	JN556038	RNA binding; RRM	mRNA stabilization/modification	2, 3, 4	[46]–[49]
CsPABP2	JN600528	RNA binding; RRMs	mRNA stabilization/modification	2, 3, 4	[46]–[51]
CsPCBP	JN600525	RNA binding; PCBP_KH domains	mRNA processing/splicing; translational activation	2, 3	[45], [61], [62]
CsTRAX	JN600526	Translin domain	RNA-induced gene silencing; DNA repair/recombination	2, 3, 4	[40], [41], [43], [44]
CsSMC	JN600522	SMC/Mnd1 domain	chromatin segregation; DNA repair/recombination	2, 3, 4	[25], [42], [44]
CsVIP2	JN600527	CCR4-NOT domain	chromatin remodeling; DNA integration	4	[50], [63]
CsRRMP1	JN600523	RNA binding; RRMs	mRNA stabilization/modification	2, 3	
CsHAP3	JN600524	DNA binding	transcription regulation	2, 3	[64]

The selected preys include a high-mobility group protein (CsHMG), two poly(A)-binding proteins designated CsPABP1 and CsPABP2, a poly(C)-binding protein with a KH domain (CsPCBP), a translin-associated factor X (CsTRAX), a structural maintenance of chromosomes domain-containing protein (CsSMC), a VirE2-interacting protein (CsVIP2), an RNA-recognition motif (RRM) protein (CsRRMP1) and a homolog of the OsHAP3A (CsHAP3) transcriptional factor (Table 1). Interestingly, all the prey proteins have either single or multiple RNA and/or DNA-binding motifs and are predicted to be nuclear. With the exception of CsRRMP1, all the identified preys have yeast, human or plant homologs with three-dimensional structure and/or biological function known (Table 1). Additional prey proteins involved in transcription regulation, including an auxin-response factor (CsARF) and a homolog of human MAF1 (CsMAF1), a negative regulator of RNA polymerase III, were isolated but will be described elsewhere.

**Figure 1:**
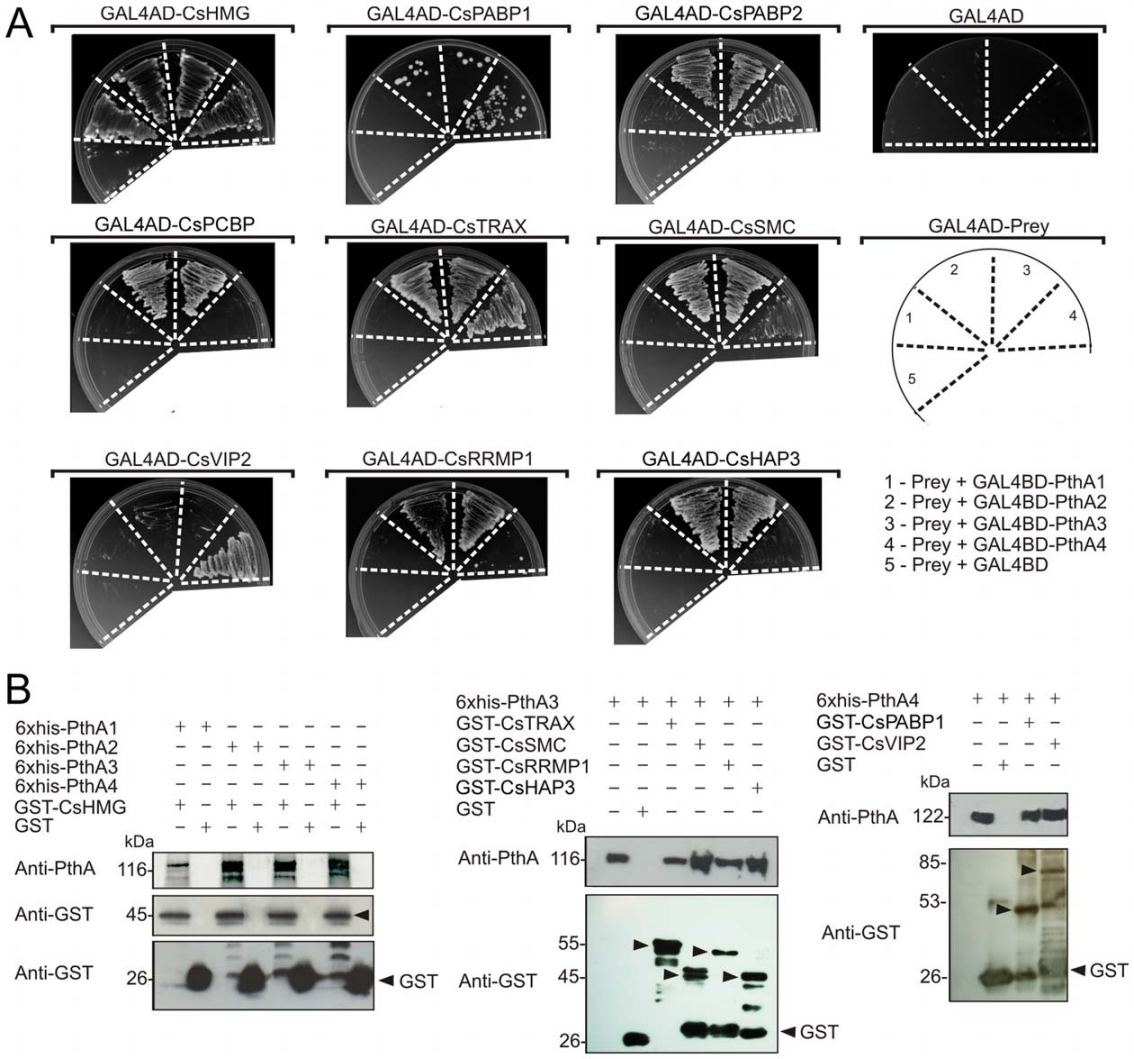
Protein-protein interactions between PthAs and citrus nuclear proteins. (A) Citrus preys fused to yeast GAL4-AD (GAL4AD-prey) or control plasmid (GAL4AD) were moved into yeast cells carrying one of the four PthA variants fused to GAL4-BD domain as shown in the diagram (1 to 4, respectively). Yeast double-transformants were grown on SC -Trp -Leu -His -Ade in the presence of 5 mM of 3AT. None of prey fusions transactivated the reporter genes when co-transformed with empty bait vector (5). The PthA baits also did not transactivate the reporter genes when co-transformed with the empty prey vector in the same growth conditions (GAL4AD). (B) Western blot detection of eluted fractions from GST pulldown assays using the purified 6xHis-PthAs 1–4 as prey and immobilized GST or GST-fusion proteins as baits. Arrows indicate bands corresponding to the expected size for the GST-fusion proteins CsHMG (∼45 kDa), CsTRAX (∼55 kDa), CsSMC (∼45 kDa), CsRRPMP1 (∼50 kDa), CsRRMP2 (∼46 kDa), CsPABP1 (∼53 kDa) and CsVIP2 (∼85 kDa) detected by the GST anti-serum. PthA proteins (∼116–122 kDa) were detected using the anti-PthA serum. Recombinant PthAs 3 and 4 were added as references in the first lanes of the gels shown in the middle and right panels, respectively.

As reported previously, some of the citrus proteins that were identified as targets of PthAs 2 and 3 also interacted with other PthA variants [16]. Thus, the identified PthA4 preys were tested for interactions with the four PthA variants in yeast two-hybrid assays. As shown in Fig. 1A, the majority of the preys interacted not only with PthA4, confirming the primary two-hybrid screening, but with PthAs 2 and 3, preferentially. Notably, CsVIP2 interacted specifically with PthA4, whereas CsPCBP, CsRRMP1 and CsHAP3 interacted with PthAs 2 and 3 under more stringent conditions (no adenine). By contrast, CsHMG was the only prey capable of interacting with all the PthA variants in the absence of adenine (Fig. 1A).

To confirm the interactions observed in yeast, GST pulldown assays were performed using one or more of the PthA variants as representative baits (Fig. 1B). For instance, CsHMG was tested against all PthAs since it showed no preference for any of the PthA variants. PthA4 was used to probe the interactions with CsPABP1 and CsVIP2, whereas CsTRAX, CsSMC, CsRRMP1 and CsHAP3 were tested against PthA3, since they interacted more strongly with this bait in the two-hybrid assays (Fig. 1A). As shown in Fig. 1B, all prey-bait interactions were confirmed by GST pulldown assays, indicating that the citrus proteins identified as PthA4-interactors are indeed novel PthA targets. The citrus proteins CsPCBP and CsPABP2 were expressed in the insoluble fraction in *E. coli* cells and thus could not be tested in the GST pulldown assays.

### Evidence for protein-protein associations among the PthA interactors

**Figure 2:**
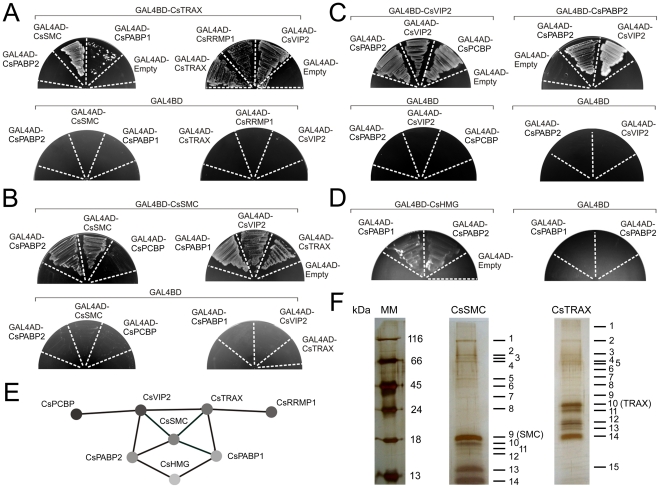
Protein-protein interactions among the PthA4 interactors detected by yeast two-hybrid and mass spectrometry. Yeast cells double-transformed with the indicated prey-bait constructs were grown in SC -Trp -Leu -His -Ade in the presence of 5 mM 3AT. (A) Positive interactions observed between CsTRAX and CsSMC, CsPABP1, CsTRAX, CsRRMP1 and CsVIP2. (B) Protein-protein interactions observed between CsSMC and CsPABP2, CsSMC, CsPABP1, CsVIP2 and CsTRAX, but not between CsSMC and CsKH. (C) Interactions of CsVIP2 with CsKH, reciprocal interactions between CsPABP2 and CsVIP2, and self interactions of CsVIP2 and CsPABP2. (D) Weak interactions between CsHMG and the poly(A)-binding proteins CsPABP2 and CsPABP1. (E) A diagram illustrating the network of interactions observed among the citrus PthA targets. (F) Silver-stained SDS polyacrylamide gels of citrus proteins trapped in cobalt beads carrying the recombinant 6xHis-tagged CsSMC or CsTRAX as baits (bands 9 and 10, respectively). Protein bands excised from the gels, indicated by the numbers, were identified by mass spectrometry (see Table 2 for details). The molecular markers (MM) are shown on the left.

Considering that the newly-identified PthA interactors are functionally related (Table 1), we tested whether they would interact with each other. As shown in Fig. 2A, a substantial number of protein-protein associations were detected relating for instance CsTRAX with CsSMC, CsPABP1, CsRRMP1 and CsVIP2. In addition CsTRAX self-interacted in yeast two-hybrid assays (Fig. 2A). Accordingly, CsSMC used as bait also interacted with CsTRAX, CsPABP1, CsPABP2 and CsVIP2, and it self-interacted (Fig. 2B), corroborating literature data [25]. CsVIP2 and CsPABP2 interacted with each other in reciprocal yeast two-hybrid assays and they also self-interacted (Fig. 2C). Moreover, CsVIP2 associated with CsPCBP, whereas CsHMG showed weak interactions with PABP1 and PABP2 (Fig. 2D).

These results pointed to a network of interactions among the PthA4 targets and suggested the existence of a citrus multiprotein complex where CsSMC would possibly function as a hub protein (Fig. 2E). To further investigate this, and considering that CsSMC and CsTRAX were the two proteins displaying higher number of protein-protein interactions, cell extracts of citrus leaves were incubated with the 6xHis-tagged CsSMC or CsTRAX immobilized in cobalt beads. Bound proteins separated by gel electrophoresis were identified by mass spectrometry (Figure 2F). Consistent with the two-hybrid data, we detected peptides corresponding to CsTRAX and a PABP 91% identical to CsPABP2 in the CsSMC sample (Table 2). Similarly, we identified CsSMC in the CsTRAX sample, but most interestingly, we found CsMAF1 and additional proteins implicated in transcriptional and translational control, such as transcription factor BTF3, Argonaute protein AGO1, translation initiation factor 5a and translation elongation factor 1-alpha, as binding partners of CsSMC and CsTRAX (Table 2). Moreover, a peptide corresponding to a citrus cyclophilin that is 81% identical to CsCYP, identified previously as an interactor of PthAs [16], was detected in the CsSMC sample (Table 2). Intriguingly, one of the citrus proteins identified by mass spectrometry associated with CsTRAX is a homolog of Bs3, a flavin monooxygenase that is induced by AvrBs3 and confers resistance against *Xanthomonas vesicatoria* strains carrying AvrBs3 [4].

**Table 2:** *Citrus sinensis* proteins identified as binding partners of CsSMC and CsTRAX by mass spectrometry.

Protein bands	Binding partners of CsSMC/CsTRAX	Accession number	Peptide sequences identified by mass spectrometry	m/z	Charge
**CsSMC**					
1,4,6–10,12	CsSMC	110848831	LRAEIANSEK	565.8135	+2
			KGYAENYEHGQVMEK	594.9451	+3
			QELTGQAQMMSQDLVR	925.9453	+2
1	Argonaute 1 (AGO1)	188428433	SLYTAGPLPFLSK	697.3930	+2
			GGVGMGSGGRGGHSGGPTR	828.8859	+2
2,3	HSP70	110877680	VEIIANDQGNR	614.8190	+2
			NQVAMNPSNTIFDAK	825.4041	+2
			IINEPTAAAIAYGLDKK	894.5037	+2
	PABP2 homolog	94441024	VAEAMEVLR	509.2771	+2
			GMPDVSMPGVGGMLPIPYGDMAAMPLR	936.7758	+3
		55396367	NMQDFPFDMGAGGMLPVPVDMGAGIPR	962.0988	+3
5	Translation elongation factor 1a	56584680	GFVASNSKDDPAR	682.3378	+2
			YYCTVIDAPGHR	726.3433	+2
			VETGVLKPGMVVTFGPSGLTTEVK	816.1167	+3
	CsMAF1	110836827	EWSETYGGSSLLETLYK	981.9786	+2
	CsTRAX	188246976	MDTMLQSVLK	583.3041	+2
			LHQLSGTALQSIAK	489.6172	+3
	PABP2 homolog	63075332	NLSESTTEEDLQK	747.3532	+2
			GSGFVAFSTPEEASRALLEMNGK	1207.5867	+2
11	Translation initiation factor 5a	21651392	DGFAEGK	362.1706	+2
			VVEVSTSK	424.7421	+2
			DDLRLPTDENLLSQIK	624.0058	+3
13	Histone H4	188254614	TLYGFGG	714.3515	+1
			TVTAMDVVYALK	663.8570	+2
	CsCYP homolog	46214048	VVVADSGELP	493.2626	+2
	RNA-binding protein	188444131	SNGGSGGERGGR	545.7521	+2
**CsTRAX**					
1,9–11	CsTRAX	188291689	LHQLSGTALQSIAK	733.9211	+2
			AEADLVAVKDQYISR	839.4460	+2
			DAFANYAGYLNELNEK	916.4340	+2
2	Argonaute 1 (AGO1)	55289153	QADAPQEALQVLDIVLR	940.0219	+2
			SGNILPGTVVDSK	643.8547	+2
		55288894	GQESENSQEAFR	691.3056	+2
3	HSP70	38053102	NALENYAYNMR	679.8090	+2
			ATAGDTHLGGEDFDNR	838.3737	+2
			NAVVTVPAYFNDSQR	840.9252	+2
4	TPR-containing protein	218827114	RIPLDFLQGEK	658.3755	+2
			MLQADQVSLAEK	666.8473	+2
			SLAQQYTWSSAVK	734.8757	+2
5	Ribosomal protein	188380114	YPLTTDSPMKNIDDK	877.4249	+2
6	Nucleosome protein	188306400	LQNLAGQHSDVLEK	776.4106	+2
7	Translation elongation factor 1a	56584680	MDATTPK	382.1881	+2
			GFVASNSKDDPAR	682.3345	+2
			YYCTVIDAPGHR	726.3430	+2
8	Protein kinase	56534189	GALSPSTAVNFALDIAR	851.9663	+2
			GMAYLHNEPNVIIHR	588.6417	+3
12	CsMAF1	110836827	INDFLDHLNLGER	778.4004	+2
			EWSETYGGSSLLETLYK	981.9799	+2
			LPECEIYSYNPDSDSDPFLEK	1259.5583	+2
13	BTF3 factor	188439148	MNVEKLMNMAGALR	805.3939	+2
14	CsSMC	110848831	LTADLQQVPALK	648.8824	+2
			QELTGQAQMMSQDLVR	917.9503	+2
15	BS3-like protein	188271719	MKEQAAGVEAIIVGAGTSGLATAACLSLQSIPYVILER	1302.0349	+3

Protein bands are numbered and they correspond to those depicted in Fig. 2F.

Taken together, these results strongly indicate that the citrus proteins identified as PthA4 interactors assemble into single or multiple protein complexes.

### CsHMG is a group B HMG homologous to AtHMGB1

Among the PthA4-interacting proteins identified (Table 1), CsHMG was the only one to interact with all the PthA variants (Fig. 1), suggesting that it is another generic target of PthAs, as the citrus importin-α [16]. In addition, CsHMG is unique in the sense that it is implicated in a variety of biological processes associated with chromatin remodeling, DNA repair and general transcriptional control [18]–[22]. Hence, CsHMG was selected for further characterization.

**Figure 3:**
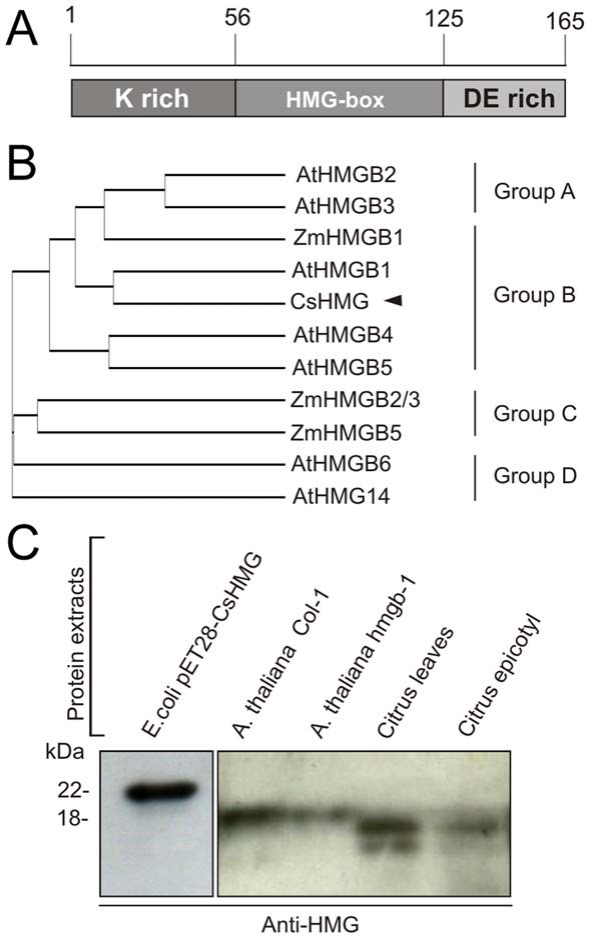
CsHMG shows identity to plant HMGBs of group B. (A) Schematic representation of the CsHMG primary structure showing its central HMG-box domain flanked by the basic K-rich N-terminal and the acidic DE-rich C-terminal. (B) Phylogenetic analysis of plant HMGB proteins showing that CsHMG belongs to group B HMGBs. (C) Western-blot detection of the recombinant 6xHis-CsHMG (∼22 kDa) made in bacteria compared to bands detected in citrus cell extracts with the expected molecular size for the endogenous CsHMG (∼16 kDa). The anti-CsHMG serum also cross-reacted with a band of similar size in the cell extracts of *A. thaliana* wild-type and heterozygous *hmg-b1* mutant. This band, which has the expected molecular weight for AtHMGB1 (∼18 kDa), is less pronounced in the heterozygous *hmgb-1* mutant, thus indicating that CsHMG is structurally related to AtHMGB1.

CsHMG is a 165 amino acid protein with a central alpha-helical HMG-box domain that is flanked by a K-rich N-terminal and a DE-rich C-terminal domain (Fig. 3A). Multiple sequence alignments and phylogenetic analysis place CsHMG into the group B of the plant HMG protein family (Fig. 3B). CsHMG is 78% identical to Arabidopsis AtHMGB1, a chromatin-associated protein that influences cell growth [17]. Thus, to confirm the identity of CsHMG to AtHMGB1, protein extracts of sweet orange leaves and epicotyls were compared to that of seedlings of *A. thaliana* wild type and *hmgb-1* heterozygous mutant [17] using an anti-CsHMG serum raised against the recombinant CsHMG. As shown in Fig. 3C, the anti-CsHMG serum detected a unique band of approximately 18 kDa in citrus epicotyls and leaves. A band of approximately 20 kDa, which corresponds to the molecular weight of AtHMGB1, was also detected in seedlings of the wild type and *hmgb-1* mutant of Arabidopsis. As expected, in the heterozygous *hmgb-1* mutant, the band is less pronounced confirming the identity of CsHMG to AtHMGB1 (Fig. 3C).

### PthA binds to CsHMG *in vivo* through its invariable LRR region

**Figure 4:**
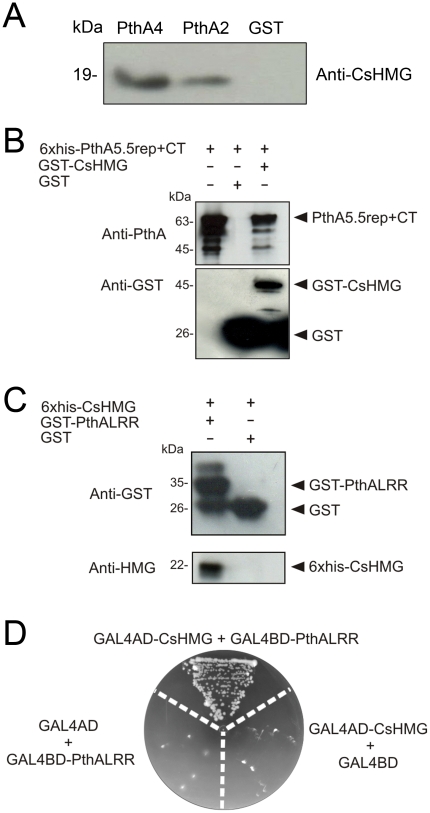
PthA binds to CsHMG *in vivo* through its invariable LRR region. (A) PthA2-GST, PthA4-GST or GST alone bound to glutathione resins were incubated with a citrus cell lisate. Bound proteins were separated by gel electrophoresis and CsHMG was detected by the anti-CsHMG serum in the PthA samples only. (B) Western blot of GST-pulldown assay of immobilized GST or GST-CsHMG as baits and purified 6xHis-PthA5.5rep+CT2 as prey. The eluted 6xHis-PthA5.5rep+CT2 (∼63 kDa) was detected by the anti-PthA serum only when GST-CsHMG was used as bait. The purified 6xHis-PthA5.5rep+CT2 was added in the first lane of the gel as reference. (C) Western blot analysis of eluted fractions of GST-pulldown assay of immobilized GST or GST-PthALRR as baits and purified 6xHis-CsHMG as prey. The eluted 6xHis-CsHMG (∼22 kDa) was detected only when GST-PthALRR was used as bait. (D) Yeast two-hybrid assay showing the interaction between CsHMG and the PthA LRR domain. Yeast double-tranformants, including controls (GAL4AD+GAL4BD-PthALRR and GAL4BD+GAL4AD-CsHMG), were grown in SC -Trp -Leu -His -Ade in the presence of 5 mM 3AT.

To confirm the interactions between PthAs and CsHMG *in vivo*, PthAs 2 and 4 fused to GST were immobilized in glutathione resins and allowed to interact with proteins from citrus cell extracts. As shown in Fig. 4A, both PthAs 2 and 4, but not GST bound to CsHMG, confirming that CsHMG is an interacting partner of PthAs.

The observation that CsHMG was the only prey to interact with the four PthA variants (Fig. 1) led us to map the PthA region required for such interactions. As the C-terminal domain of PthAs is the least variable region among the PthA variants, and that this region includes a leucine-rich repeat (LRR) that is invariable among all PthAs [16], we tested whether the C-terminal would account for the interaction with CsHMG. GST pulldown assays were performed with PthA2 constructs carrying the entire C-terminal domain or the LRR alone. Both the entire C-terminal domain and the LRR alone interacted with CsHMG, indicating that the invariable LRR is sufficient for the interaction (Fig. 4B and C). This result was confirmed by a two hybrid assay which shows that the LRR interacts with CsHMG in yeast (Fig. 4D).

### CsHMG binds DNA and poly(U) RNA

**Figure 5:**
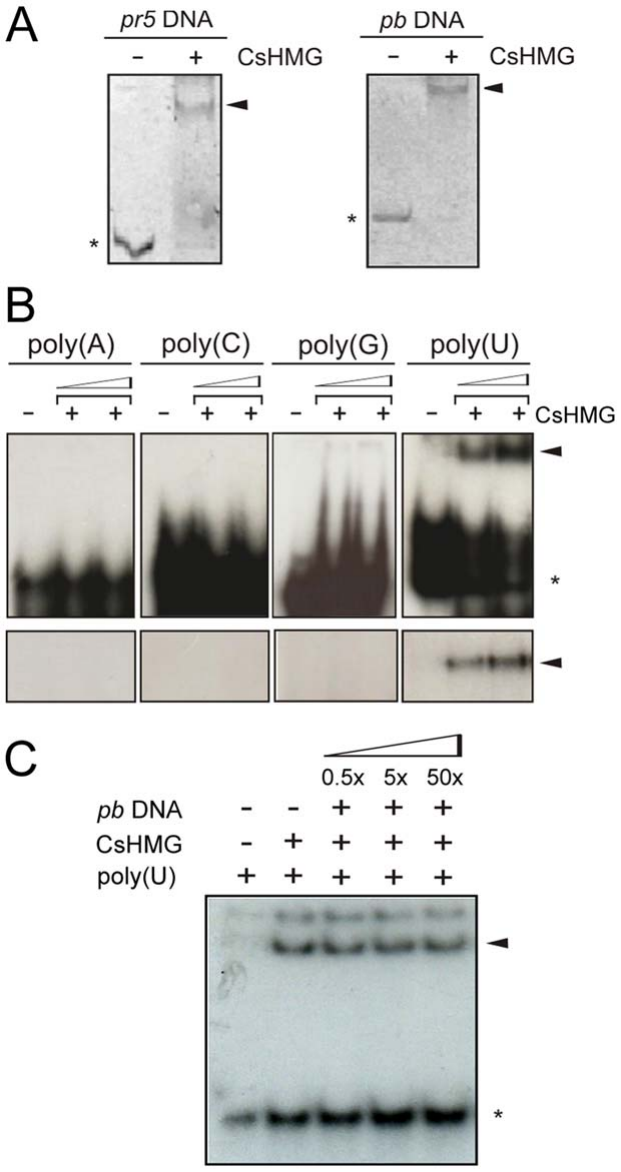
CsHMG shows DNA and RNA-binding activities in vitro. (A) EMSA using the recombinant 6xHis-CsHMG protein (5 µg) and DNA probes (200 ng) derived from the citrus *pr5* promoter and the multiple-cloning site of the pBluescript vector. The DNA-protein complexes and free probes detected by ethidium bromide staining are indicated by the arrows and asterisks, respectively. (B) Upper panel, EMSA using ^32^P-labelled single strand RNA probes at a final concentration of 12.5 nM and increased amounts of purified CsHMG (0.1 and 0.5 µg). Shifted bands corresponding to CsHMG:RNA complexes observed with the poly(U) RNA and the free probes are indicated by the arrow and asterisk, respectively. Bottom panel, SDS-PAGE of UV-crosslink EMSA showing the selective binding of CsHMG to the poly(U) RNA probe (arrow). (C) EMSA using the ^32^P-labelled poly(U) RNA as probe at a final concentration of 12.5 nM, 100 ng of CsHMG and increasing amounts of the double strand DNA (multiple-cloning site of the pBluescript vector) as competitor. Shifted bands corresponding to CsHMG:RNA complexes and the free probe are indicated by the arrow and asterisk, respectively.

HMGBs are highly abundant chromosomal proteins known to bind DNA in a non-specific manner [26], [27]. Thus, we examined the ability of the recombinant CsHMG to bind DNA in gel-shift assays by testing its interaction with two unrelated double-strand DNA probes, one derived from the citrus *pr*5 promoter [28] and another derived from a bacterial cloning vector. As shown in Fig. 5A, CsHMG bound to the two DNA probes, indicating that the citrus protein does not display DNA sequence specificity, a general feature of HMGs. Although this result is in line with the literature data and with our observation that CsHMG made in *E. coli* co-purifies with bacterial DNA (not shown), we also noticed that the removal all traces of nucleic acids from our recombinant CsHMG preparations required an RNase treatment, which indicated that CsHMG had affinity for RNA as well. To test this hypothesis, we performed gel-shift assays to probe the binding of CsHMG to single-strand RNAs. Surprisingly, we found that CsHMG not only binds to single-strand RNA *in vitro* but it shows specificity to poly(U) RNAs (Fig. 5B, upper panel). The specific binding of CsHMG to poly(U) RNA was further confirmed by an UV-crosslinking gel-shift assay (Fig. 5B, bottom panel).

Next, to test whether CsHMG would preferentially bind to poly(U) RNA or DNA, we performed a competition gel-shift assay using a double strand DNA as competitor. The results shown in Fig. 5C suggest that CsHMG binds to the poly(U) RNA preferentially.

### CsHMG selectively binds to poly(U) RNA through its HMG-box domain

**Figure 6:**
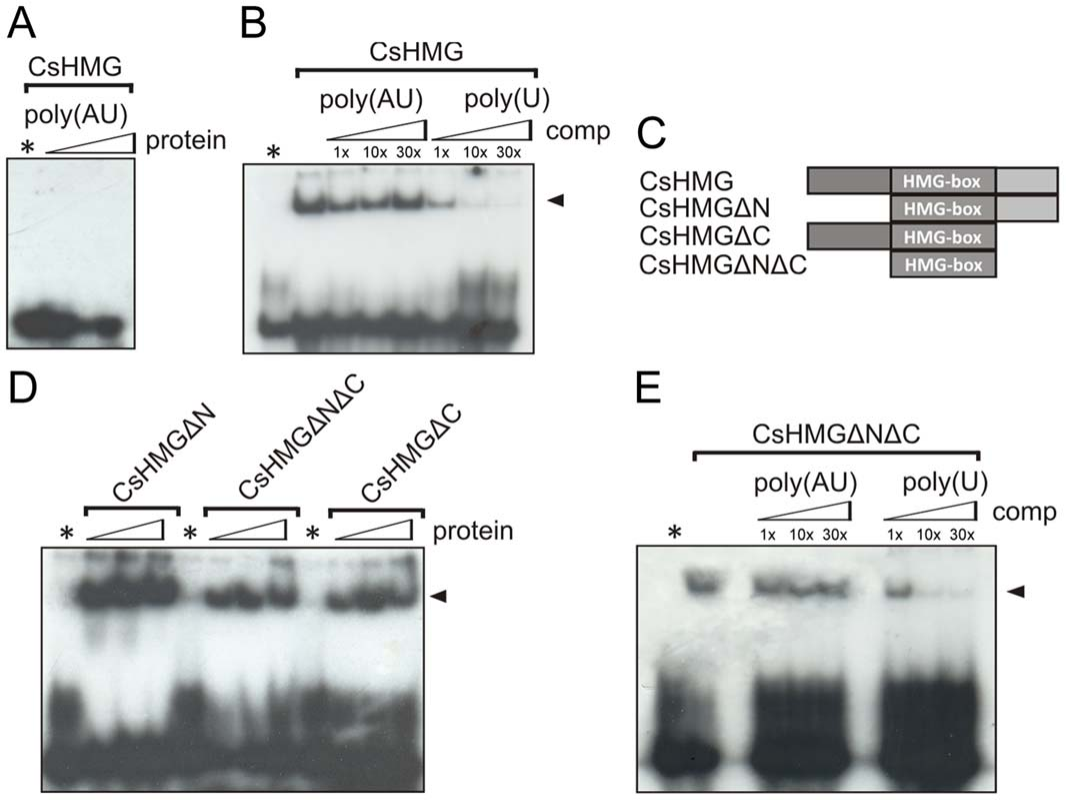
CsHMG selectively binds to poly(U) RNAs through its HMG-box domain. (A) EMSA showing that CsHMG (0.5 and 1.0 µg) does not bind to the AU-rich RNA probe (5′- UUAUUAUUUAUUUAUUUAUU-3′). (B) Competition EMSA showing that the AU-rich RNA probe at 1, 10 and 30 times molar excess does not compete with the poly(U) probe for the binding to CsHMG, as opposed to the cold poly(U) probe. (C) Schematic representation of the truncated versions of CsHMG used in the experiments depicted in D and E. (D) All CsHMG truncations were capable of binding to the poly(U) RNA probe. (E) Competition EMSA showing that the AU-rich RNA probe at 1, 10 and 30 times molar excess does not compete with the poly(U) probe for the binding to the HMG-box (CsHMGΔNΔC), as opposed to the cold poly(U) probe, indicating that the HMG-box of CsHMG is sufficient to confer the poly(U) RNA-binding specificity.

To test whether CsHMG is capable of binding to non-contiguous U-rich sequences, an AU-rich probe was used in the gel-shift assays. As shown on Fig. 6A, no shifted bands were detected when an AU-rich RNA was used as a probe at different concentrations. Moreover, the AU-rich probe could not compete with the poly(U) probe in a competition gel-shift assay (Fig. 6B), indicating that the RNA-binding activity of CsHMG is specific towards contiguous U-rich sequences.

To map the CsHMG region responsible for the RNA interaction, three truncated forms of CsHMG, CsHMGΔN (no N-terminal), CsHMGΔC (no C-terminal) and CsHMGΔNΔC (only the HMG-box) were employed in gel-shift assays (Fig. 6C). All truncations were capable of binding the poly(U) probe (Fig. 6D), indicating that the HMG-box domain alone is sufficient for the RNA-binding activity of CsHMG and to confer the specificity to poly(U) RNA. In fact, CsHMGΔNΔC retained its selective binding to poly(U) even when the poly(AU) probe was used as a competitor in the gel-shift assay (Fig. 6E).

### PthA4 forms higher molecular weight complexes with poly(U) RNA in the presence of CsHMG

**Figure 7:**
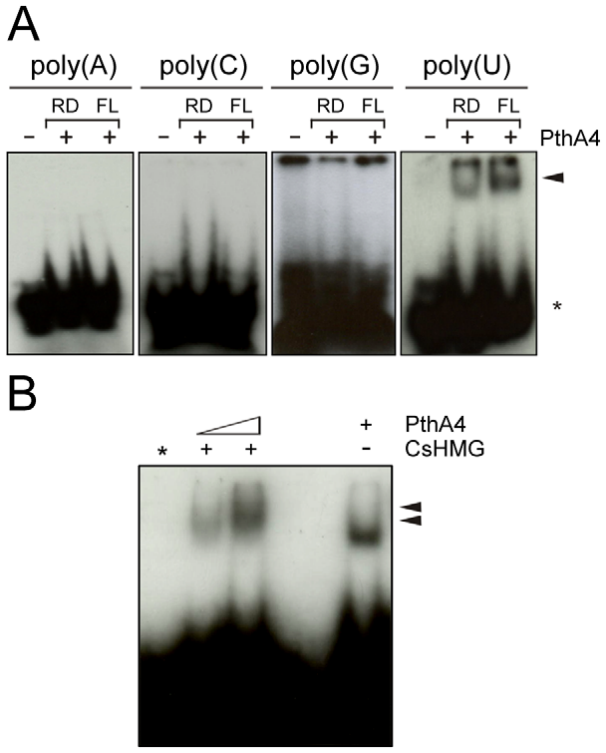
PthA4 and its repetitive domain bind to poly(U) RNA. (A) EMSA using ^32^P-labelled single strand RNA probes at a final concentration of 12.5 nM and 100 ng of the purified full-length (FL) PthA4 or its internal repetitive domain (RD). Shifted bands corresponding to the RNA:protein complexes and the free probes are indicated by the arrow and asterisks, respectively. (B) EMSA using the ^32^P-labelled single strand poly(U) probe and the recombinant full length PthA4 and CsHMG. PthA4 forms higher molecular weight bands in the presence of CsHMG (arrows). The CsHMG-poly(U) complex is not resolved from the free probe (*) in the 6% polyacrylamide gel.

Considering that (i) CsHMG selectively binds to poly(U) RNA, (ii) CsHMG and PthA4 interact with two poly(A)-binding proteins, (iii)- most of the PthA4 interactors have RNA recognition motifs and are implicated in mRNA stabilization and processing, (iv) recombinant PthAs made in *E. coli* co-purifies with RNA, and (v) the DNA-binding domain of PthAs is predicted to fold like a pentatricopeptide repeat (PPR) [28], a protein domain that recognizes U-rich sequences and plays roles in mRNA stabilization and editing [29]–[31], we tested whether PthA4 would interact with RNA molecules. Surprisingly, we found that PthA4 and its internal repetitive DNA-binding domain were capable of selectively binding to poly(U) RNA in gel shift assays (Fig. 7A). Furthermore, in the presence of CsHMG, PthA4 formed higher molecular weight complexes with poly(U) RNA (Fig. 7B), suggesting formation of a ternary complex.

## Discussion

Despite the abundant genetic data showing that bacterial TAL effectors function as transcriptional activators in host cells, little is known about the molecular mechanism through which they act. Previously, we identified CsCYP, CsTDX and CsUEV/CsUBC13 heterodimer as targets of PthAs. These proteins, which are associated with the basal transcription machinery, interact with each other and affect DNA repair [16]. Here, we present evidence suggesting that PthAs target a novel nuclear multiprotein complex whose components are implicated in chromatin remodeling and repair, transcription regulation, mRNA stabilization/modification and translational control (Tables 1 and 2).

A component of this protein complex, CsHMG, shows selective binding to poly(U) RNA. Although homologs of CsHMG in animals participate in a variety of biological processes associated with sex determination, DNA repair and cancer [20], [21], [32], [33], the roles played by CsHMG homologs in plants are less clear [34], [35]. Plant HMGBs similar to CsHMG have been suggested to promote the assembly of nucleoprotein complex involved in transcriptional control [19]. In maize, HMGB1 interacts with transcription factors of the bZip and Dof families and promotes Dof DNA binding through its acidic C-terminal domain [19], [36]. In mammals, HMGB1 interacts with the N-terminus of the TATA-binding protein (TBP) to form a stable ternary complex with TBP and the TATA element to repress transcription [37], [38]. Interestingly, the basal transcriptional factor TFIIA was shown to bind TBP and displace HMGB1 from the inhibitory HMGB1/TBP/TATA complex, allowing transcription initiation [39]. Thus, it is possible that PthAs could play a similar role as TFIIA. This idea is in line with the observation that PthAs bind to sites at or close to predicted TATA-box elements of citrus promoters (unpublished results).

The fact that CsHMG specifically binds to poly(U) RNA indicates however that it may play other roles beyond that of an architectural DNA-binding factor. Surprisingly, mammalian HMGB1 was shown to bind branched rRNAs and inhibit RNA cleavage by a ribozyme, which implicates HMGBs in RNA processing [23]. In this respect, it is worth noting that human TRAX, which is 37% identical do CsTRAX, is a component of the RNA-induced silencing complex (RISC) which displays endoribonuclease activity [40], [41]. In yeast, TRAX binds to C1D, a SMC-interacting protein that is essential for the repair of double-strand DNA breaks [42]–[44]. Additionally, the human homolog of CsPCBP was shown to bind conserved UC-rich motifs within the 3′-untranslated region of an mRNA [45]. Hence, the putative citrus multiprotein complex identified here is thought to play roles in DNA repair and RNA stabilization and/or processing mechanisms.

**Figure 8:**
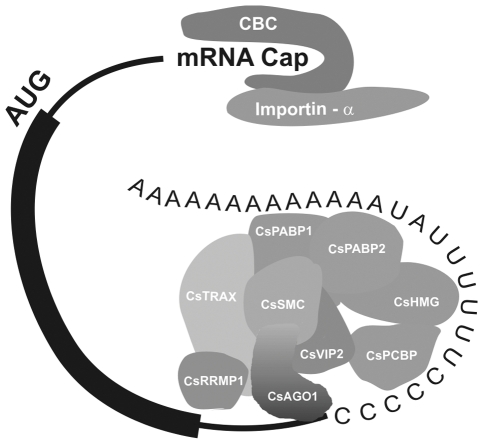
Schematic model of the citrus multiprotein complex comprising the PthA4-interacting partners. Protein-protein and protein-RNA contacts involving the PthA4 interactors based on the yeast two-hybrid, GST-pulldown and gel-shift assays described here, and literature data. The citrus multiprotein complex is reminiscent of that of mammalian miRISC involved in miRNA-mediated deadenylation [51]. Importin-α, which interacts with all PthA variants [16] is also a component of the cap-binding complex (CBC) which inhibits mRNA deadenylation when in the presence of a poly(A)-specific ribonucleases [55]–[58]. It is suggested that by interacting with such proteins and with poly(U) RNA (not necessarily simultaneously), PthA proteins may displace some of the components of this complex thought to promote deadenylation and mRNA decay and thus increase mRNA stabilization and translation initiation. U-rich sequences found in both 5′ and 3′ ends of mRNAs could represent binding sites of CsHMG and PthA4.

Several lines of evidence support this idea. Firstly, some of the protein-protein interactions identified by two-hybrid, relating for instance CsSMC with CsTRAX and CsPABP2, were confirmed by mass spectrometry analysis (Table 2). CsPABP1 and CsPABP2 are, respectively, homologous to human PABPN and PABPC, which function as mRNA stabilizing and translation initiation factors [46], [47]. Notably, translation initiation and elongation factors, as well as a homolog of tobacco AGO1 [48], were identified as binding partners of CsSMC and CsTRAX (Table 2). Furthermore, human PABPN binds to PABPC in the presence of RNA and to RRM proteins similar to CsRRMP1 in a protein complex involved in mRNA turnover [49], [50]. Most importantly however, mammalian PABPC was recently shown to be recruited by RISC and to associate with CAF1/CCR4-NOT deadenylases in a multiprotein complex that regulates gene silencing through a miRNA-mediated mRNA deadenylation [51], [52]. Thus, considering that TRAX is a component of RISC [40], [41], AGO1 was found associated with CsSMC and CsTRAX (Table 2), and CsVIP2 is a CCR4-NOT domain protein, we propose a model (Fig. 8) in which many the PthA4 interactors identified here are components of a multiprotein complex similar to the mammalian miRISC involved in miRNA-mediated deadenylation [51]. In this model, CsHMG and CsPCBP would bind to UC-rich sequences in the 3′ end of the mRNA [45], whereas PABP1 and PABP2 would attach to the adjacent poly(A) tail creating a scaffold for the assembly of CsSMC, CsTRAX, CsVIP2, CsRRMP1 and AGO1 (Fig. 8). The fact that PthA4 selectively binds poly(U) RNA, apparently forming a ternary complex with CsHMG, is also interesting. Even though not compatible with the TAL code [7], [8], it is worth noting that the DNA-binding domain of PthA, and related TAL effectors, shows a superhelical structure though to be similar to that of PPR domains [28], [53], [54] involved in the recognition of U-rich sequences in 5′ and 3′mRNA termini [29]–[31].

Although it remains to be demonstrated, if the citrus protein complex is the equivalent of the mammalian miRISC involved in gene silencing [51], it is possible that by targeting such a complex, PthAs might inhibit mRNA deadenylation and decay, thus increasing mRNA stability for the pioneer round of translation. In line with this idea, it is interesting to note that importin-α, a strong PthA interactor [16], have additional roles besides the nucleocytoplasmic transport. Importin-α, together with importin-β, were shown to interact with the cap-binding complex (CBC), affect splicing, 3′-end formation and to inhibit mRNA deadenylation [55]–[58].

Finally, the identification of CsSMC as a hub in the PthA4 interactome is in agreement with recent data showing that independently evolved effectors converge onto hubs as common targets [59]. Surprisingly, most of these hubs associate with proteins controlling RNA binding/translation, DNA binding/chromatin remodeling/transcription and ubiquitination [59]. Although the Arabidopsis homolog of CsSMC was not identified as a target of *Pseudomonas* and *Hyaloperonospora* effectors, CsSMC is related to SKIP-interacting protein 30, a rice hub [60].

Taken together, our data suggest that PthA proteins target a novel citrus multiprotein complex involved in mRNA stabilization and processing associated with translational control.
