# Correction: The TAL Effector PthA4 Interacts with Nuclear Factors Involved in RNA-Dependent Processes Including a HMG Protein That Selectively Binds Poly(U) RNA

**DOI:** 10.1371/journal.pone.0134818

**Published:** 2015-07-31

**Authors:** Tiago Antonio de Souza, Adriana Santos Soprano, Nayara Patricia Vieira de Lira, Alexandre José Christino Quaresma, Bianca Alves Pauletti, Adriana Franco Paes Leme, Celso Eduardo Benedetti

As a result of an error during figure preparation, Fig 1B in this article includes an incorrect panel; the right-hand panel of Fig 1B is also included in Fig 1D of the following article:

Santos Soprano A, Abe VY, Costa Smetana JH and Benedetti CE: Citrus MAF1, a Repressor of RNA Polymerase III, Binds the Xanthomonas citri Canker Elicitor PthA4 and Suppresses Citrus Canker Development. Plant Physiology http://dx.doi.org/10.1104/pp.113.224642.

**Fig 1:**
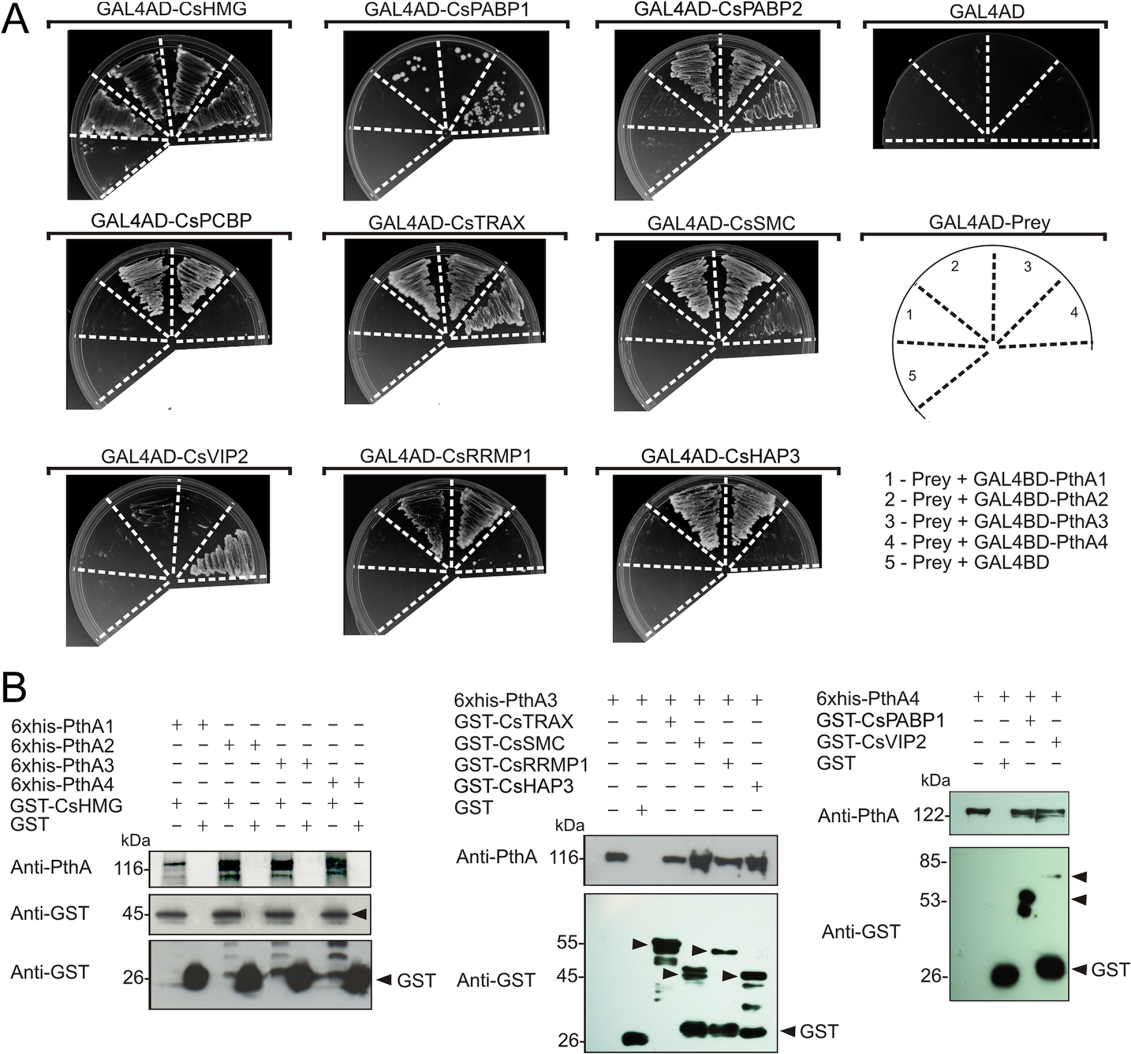
Protein-protein interactions between PthAs and citrus nuclear proteins. (A) Citrus preys fused to yeast GAL4-AD (GAL4AD-prey) or control plasmid (GAL4AD) were moved into yeast cells carrying one of the four PthA variants fused to GAL4-BD domain as shown in the diagram (1 to 4, respectively). Yeast double-transformants were grown on SC-Trp-Leu-His-Ade in the presence of 5 mM of 3AT. None of prey fusions transactivated the reporter genes when co-transformed with empty bait vector (5). The PthA baits also did not transactivate the reporter genes when co-transformed with the empty prey vector in the same growth conditions (GAL4AD). (B) Western blot detection of eluted fractions from GST pulldown assays using the purified 6xHis-PthAs 1–4 as prey and immobilized GST or GST-fusion proteins as baits. Arrows indicate bands corresponding to the expected size for the GST-fusion proteins CsHMG (~45 kDa), CsTRAX (~55 kDa), CsSMC (~45 kDa), CsRRPMP1 (~50 kDa), CsRRMP2 (~46 kDa), CsPABP1 (~53 kDa) and CsVIP2 (~85 kDa) detected by the GST anti-serum. PthA proteins (~116–122 kDa) were detected using the anti-PthA serum. Recombinant PthAs 3 and 4 were added as references in the first lanes of the gels shown in the middle and right panels, respectively.

In the published figure, the third lane along shows CsMAF1 protein–as correctly denoted in the *Plant Physiology* article–but is incorrectly labelled as CsPABP1 protein in the *PLOS ONE* article. The authors would like to apologize for the error, which arose when the wrong lane was removed from the blot during preparation of the figure. The authors have repeated the GST-pulldown experiments with freshly prepared recombinant protein samples, and now provide a corrected Fig 1 that includes the blots obtained from the new experiments. Raw, uncropped blot images for both the new experiment and the original experiment are included as Supporting Information files. This correction does not affect the conclusions of the paper.

## Supporting Information

S1 File Original experiment images: raw uncropped blots, SDS-PAGE gel, and Coomassie blue stained PVDF membrane. (ZIP)

S2 File Repeated experiment images: raw uncropped blots and SDS-PAGE gel. (ZIP)
