# Supplementary figures and images for: Correction: The TAL Effector PthA4 Interacts with Nuclear Factors Involved in RNA-Dependent Processes Including a HMG Protein That Selectively Binds Poly(U) RNA
Source: PLoS One. 2015 Jul 31;10(7):e0134818. doi: 10.1371/journal.pone.0134818 (PMC4521785; doi:10.1371/journal.pone.0134818)

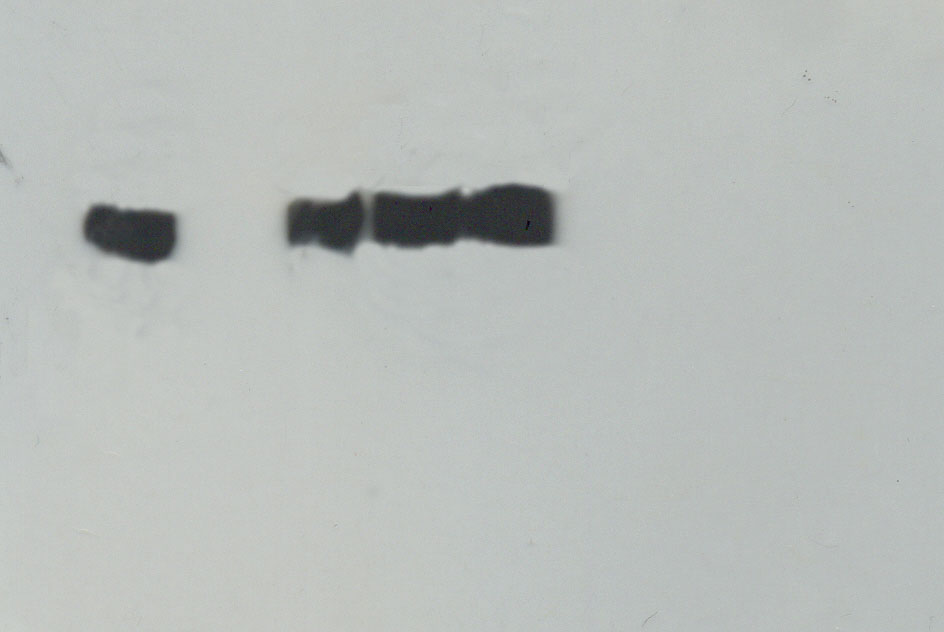

Supplement: S1 File — (ZIP) [file pone.0134818.s001.zip › Anti Ptha (4)_Maf_VIP2_PabpN 1 min.jpg]

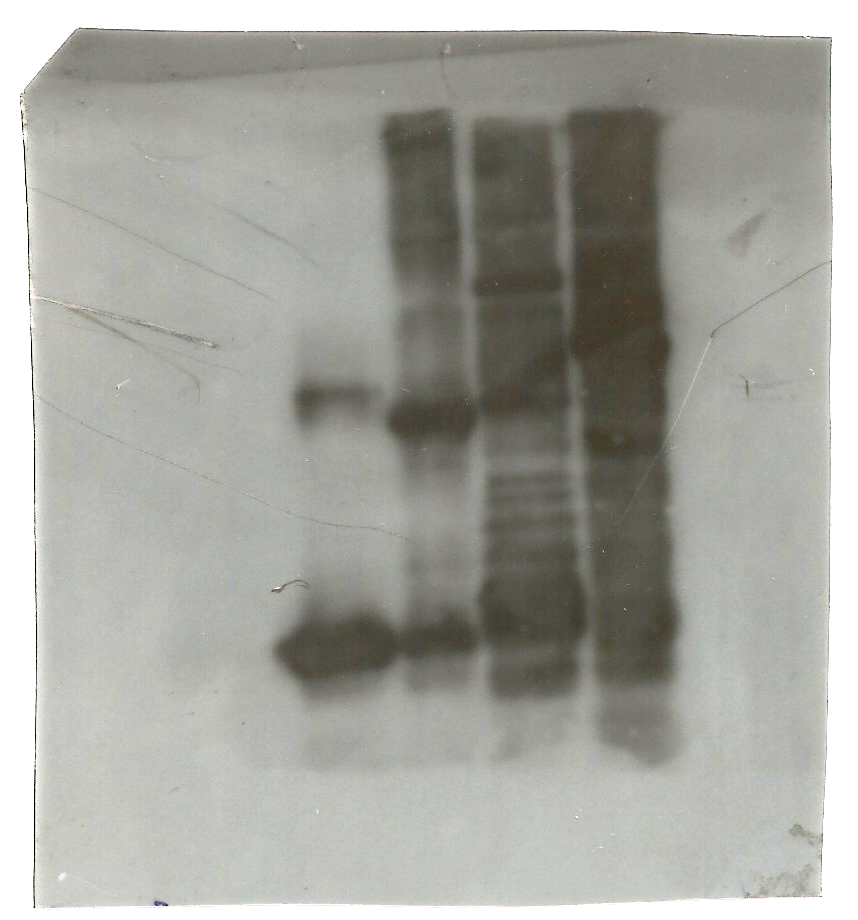

Supplement: S1 File — (ZIP) [file pone.0134818.s001.zip › Image Fig1B-2.tif]

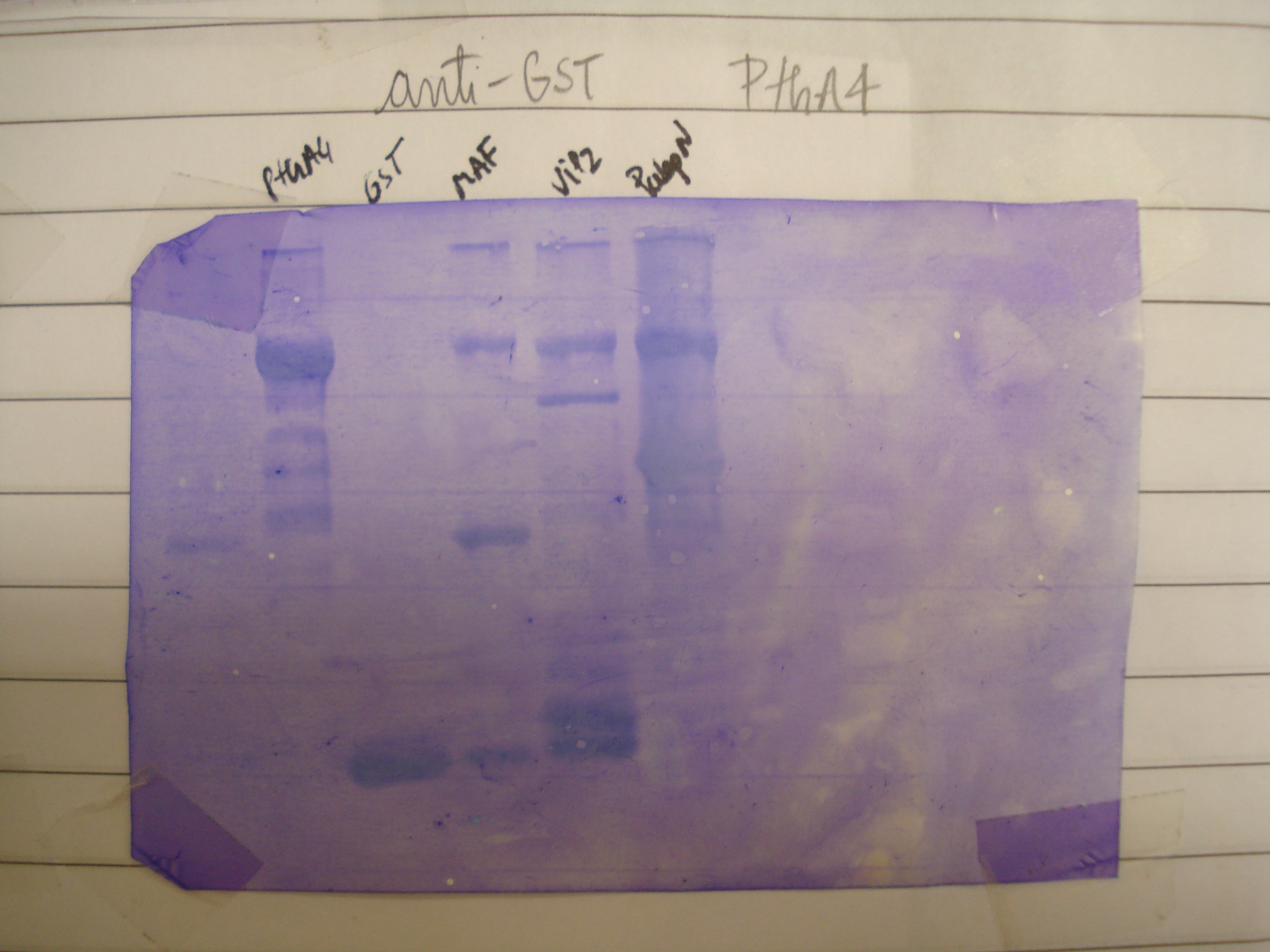

Supplement: S1 File — (ZIP) [file pone.0134818.s001.zip › Membrane.jpg]

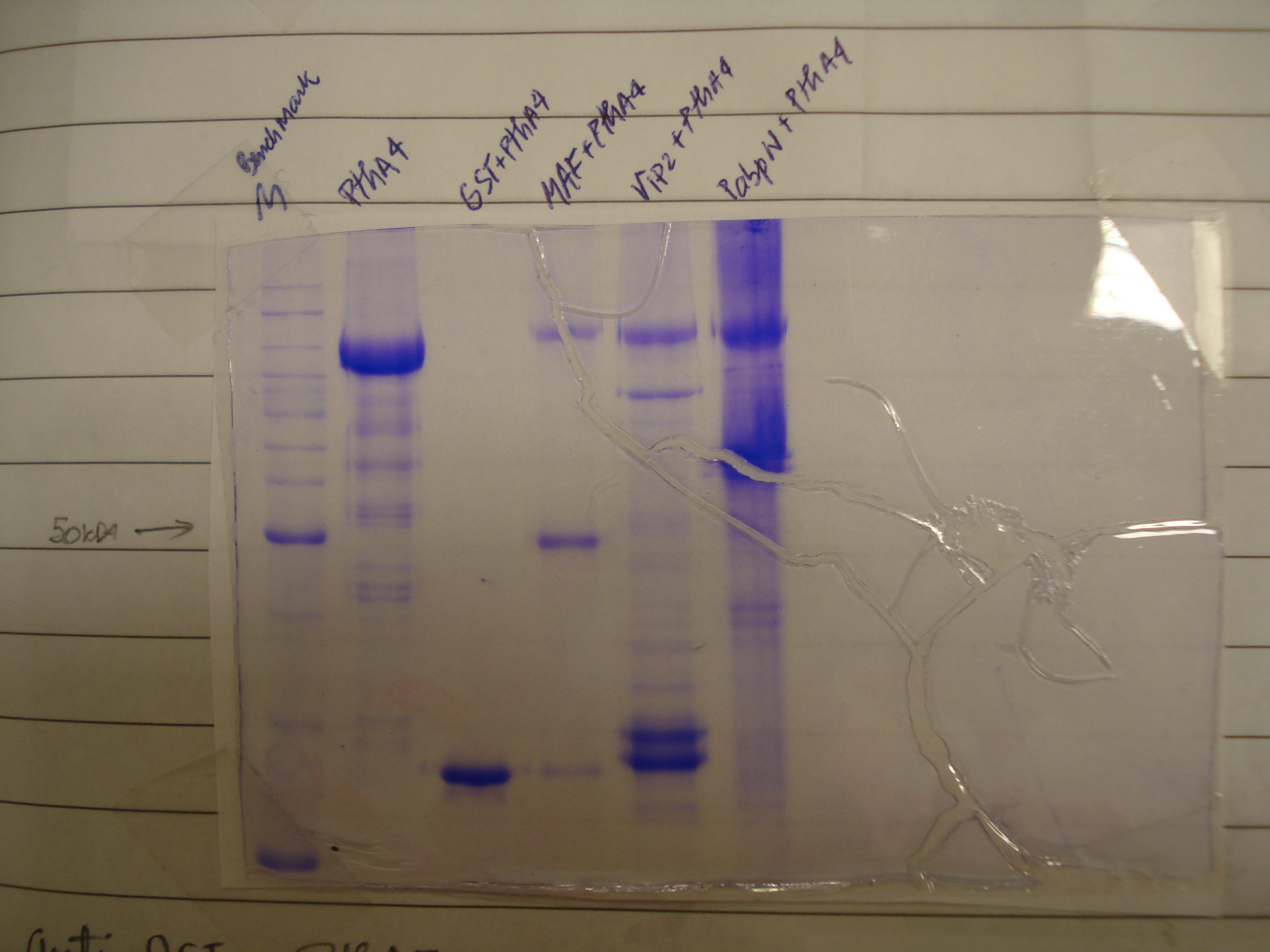

Supplement: S1 File — (ZIP) [file pone.0134818.s001.zip › SDSPAGE (1).jpg]

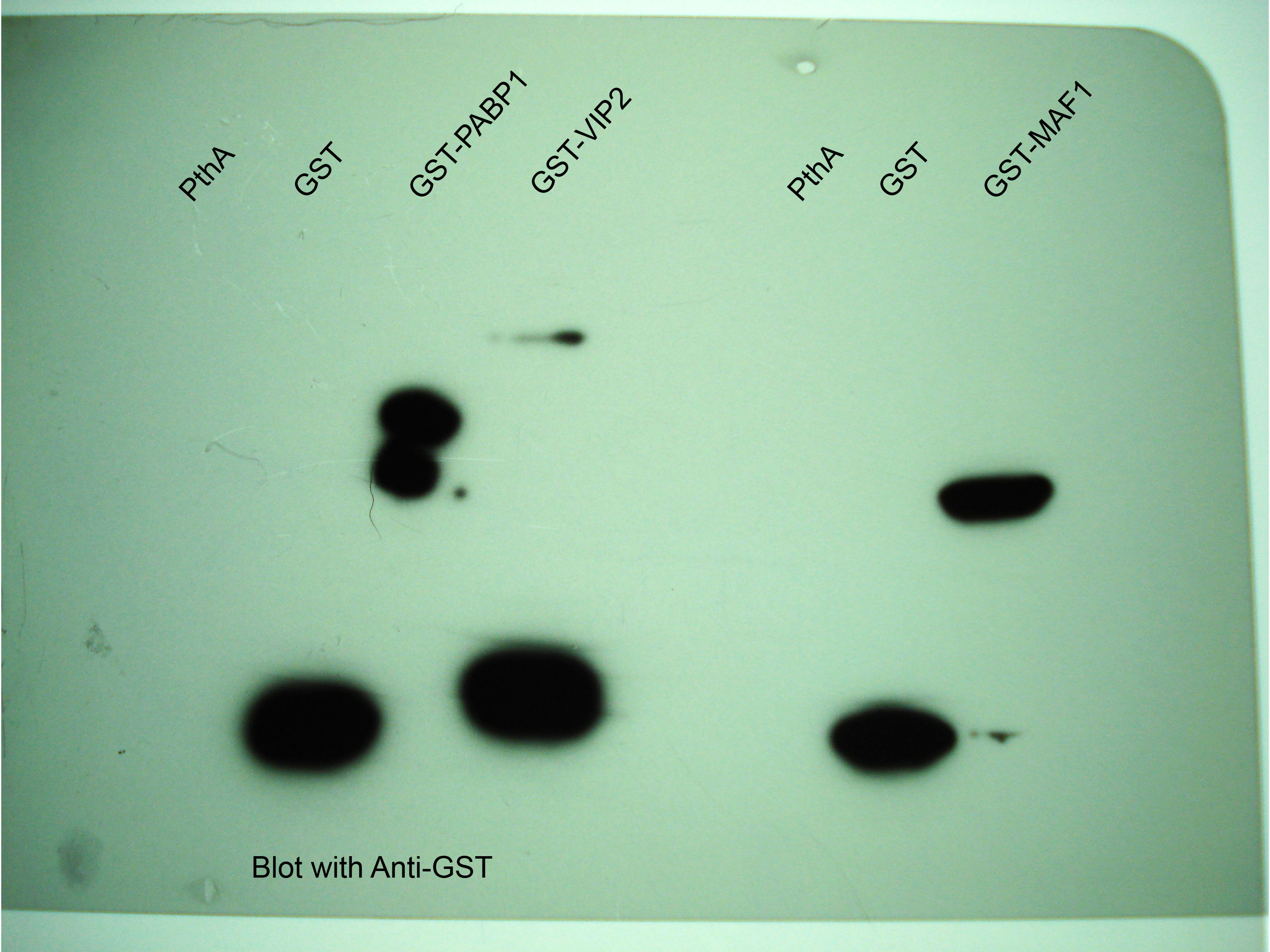

Supplement: S2 File — (ZIP) [file pone.0134818.s002.zip › Blot Anti-GST.jpg]

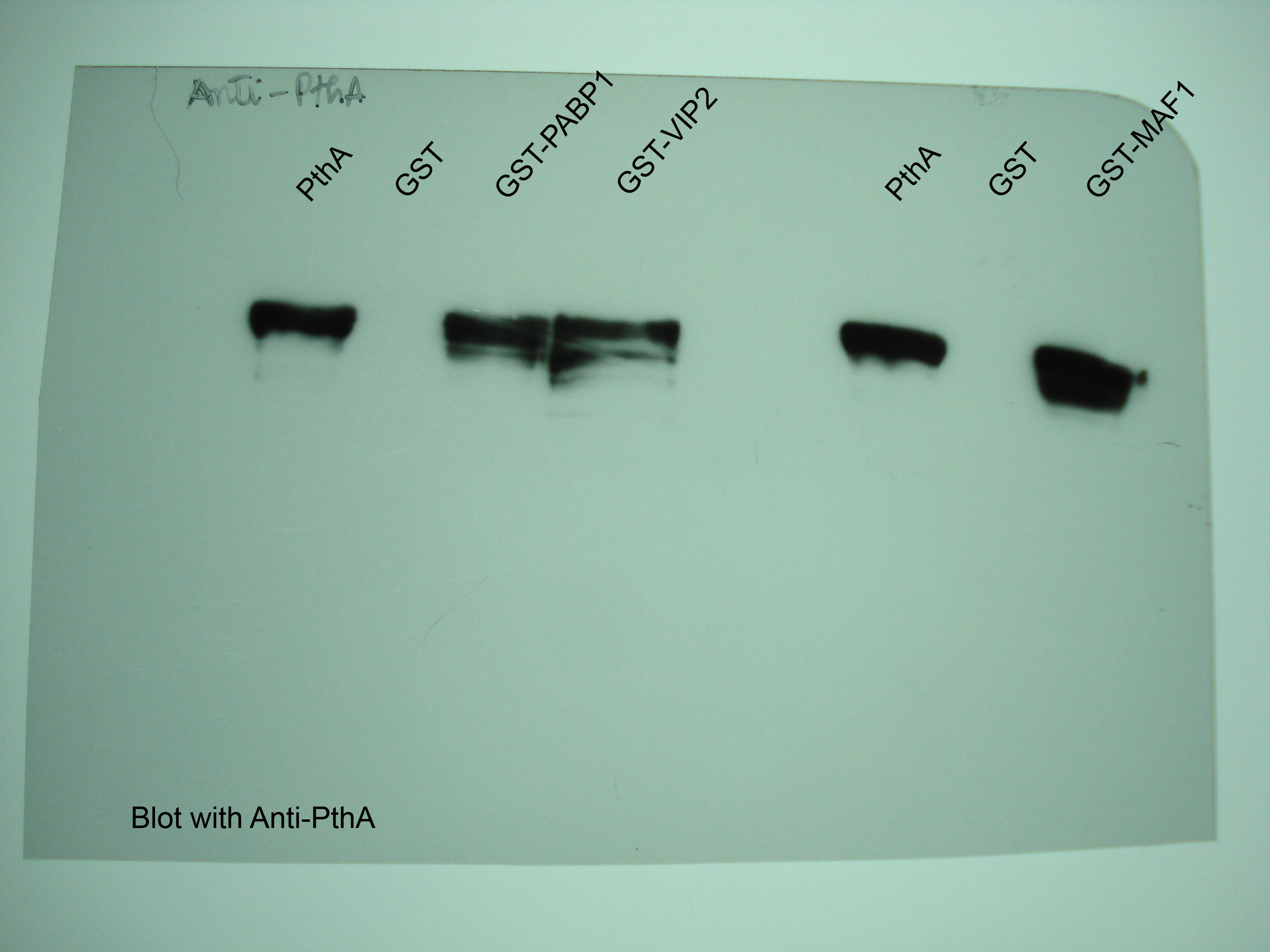

Supplement: S2 File — (ZIP) [file pone.0134818.s002.zip › Blot Anti-PthA.jpg]

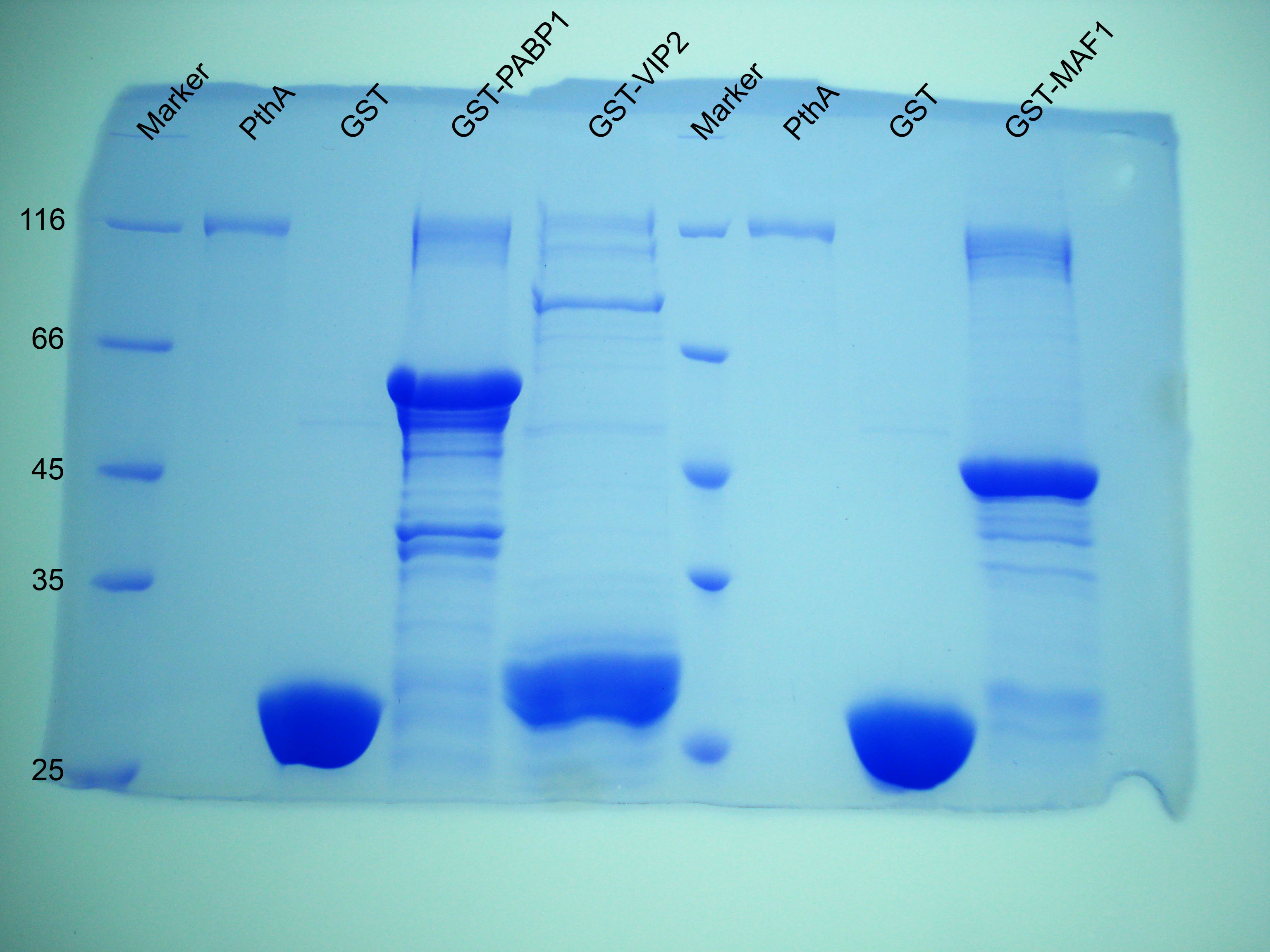

Supplement: S2 File — (ZIP) [file pone.0134818.s002.zip › SDS-PAGE.jpg]
